# Gambling-Related Distortions and Problem Gambling in Adolescents: A Model to Explain Mechanisms and Develop Interventions

**DOI:** 10.3389/fpsyg.2017.02243

**Published:** 2018-01-05

**Authors:** Maria Anna Donati, Francesca Chiesi, Adriana Iozzi, Antonella Manfredi, Fabrizio Fagni, Caterina Primi

**Affiliations:** ^1^Department of Neurofarba, University of Florence, Florence, Italy; ^2^UFC SerD Zona 1 Firenze, Azienda USL Toscana Centro, Florence, Italy; ^3^Area Dipendenze, Azienda USL Toscana Centro, Florence, Italy; ^4^UFC SerD Pistoia-Valdinievole, Azienda USL Toscana Centro, Florence, Italy

**Keywords:** gambling, prevention, adolescents, gambling-related cognitive distortions, mindware, gambler’s fallacy, superstitious thinking

## Abstract

Although a number of gambling preventive initiatives have been realized with adolescents, many of them have been developed in absence of a clear and explicitly described theoretical model. The present work was aimed to analyze the adequacy of a model to explain gambling behavior referring to *gambling-related cognitive distortions* (Study 1), and to verify the effectiveness of a preventive intervention developed on the basis of this model (Study 2). Following dual-process theories on cognitive functioning, in Study 1 we tested a model in which *mindware gap*, i.e., susceptibility to the gambler’s fallacy, and *contaminated mindware*, i.e., superstitious thinking, were the antecedents of gambling-related cognitive distortions that, in turn, affect gambling frequency and problem gambling. Participants were 306 male adolescents (*M*_age_ = 17.2 years). A path analysis indicated that cognitive distortions have a mediating role in the relationship that links probabilistic reasoning fallacy and superstitious thinking with problem gambling. Following these findings, in Study 2 we developed a school-based intervention aimed to reduce gambling-related cognitive distortions acting on the above cited *mindware* problems. A pre- and post-test design – with a 6 months follow-up – was performed with 34 male adolescents (*M*_age_ = 16.8), randomly assigned to two groups (Training and No Training), and their baseline equivalence was verified. A Mixed 2 × 2 ANOVA attested a significant Time X Group interaction, indicating a significant reduction of the cognitive distortions from pre-test to post-test only in the Training group. The follow-up attested to the stability of the training effects and the reduction of gambling frequency over time. These findings suggest that prevention strategies should address *mindware* problems, which can be considered as predictors of gambling-related cognitive distortions.

## Introduction

Despite the restrictions to gamble for youth, prevalence studies report that a large number of adolescents are involved in gambling activities and that they are at higher risk for developing gambling problems compared to adults (see Blinn-Pike et al., 2010; Volberg et al., 2010, for reviews). There are alarming data as initiation of gambling at an early age is associated with a higher risk of more severe gambling problems in adulthood (Johansson et al., 2009; Granero et al., 2014). Due to the potential negative consequences derived from gambling, prevention of problem gambling among adolescents has increasingly become an important area of concern in research and practice. For this reason, several educational initiatives have been realized (see Ladouceur et al., 2013; St-Pierre et al., 2015; Keen et al., 2016, for reviews). However, many of them have been developed in absence of an explicitly described theoretical model (Ladouceur et al., 2013; St-Pierre et al., 2015) and, even when a theoretical model has been proposed, it was adapted from other addictions’ prevention approaches or it is often unclear how the theory was used in the program development (St-Pierre et al., 2015).

Following this premise, the goal of the present work was to fill this gap through two studies. In Study 1, we aimed to test a theoretically grounded model to explain gambling frequency and problem gambling referring to *gambling-related cognitive distortions,* i.e., a wide array of mistaken beliefs and perceptions about gambling (Raylu and Oei, 2004; Johansson et al., 2009). Then, in Study 2, we aimed to develop and verify the effectiveness of a preventive intervention focused on *gambling-related cognitive distortions* based on the model tested in Study 1.

## Study 1

Referring to research with adults, dual-process theories on cognitive functioning have been used to explain cognitive failure that leads to persistent gambling behavior (Toplak et al., 2007). These theories distinguish between autonomous sets of systems (rapid, automatic, parallel, and heuristic) and analytic cognitive processes (slow, under control, serial, and rule-based) (see Stanovich, 2004, for a review). Toplak et al. (2007) used this model to explain how people tend to be irrational while gambling. Particularly, they considered problems regarding *mindware* (Perkins, 1995), defined as the rules, procedures, and strategies derived from past learning experiences and available for explicit retrieval. The authors stated that mindware problems can arise when there is a *mindware gap* or in the case of *contaminated mindware*. Specifically, there is a *mindware gap* when the appropriate rules, procedures, and strategies are lacking, while a *contaminated mindware* verifies when the employed mindware is not helpful in the specific situation. Referring to gambling, to operationalize the *mindware gap*, they referred to probabilistic reasoning ability in a variety of heuristic and bias problems and they proposed the disposition to believe in paranormal events, superstition, and luck to operationalize the *contaminated mindware*.

On one hand, a *mindware gap* in probabilistic reasoning, intended as the ability to draw conclusions about the likelihood of events based on available information or personal knowledge or beliefs, could have an important role in gambling. Indeed, it has been suggested that misunderstanding of probability can lead to irrational thoughts and behaviors related to gambling, such as chasing or obtaining false contingencies (Raylu and Oei, 2004). As reviewed by Goodie and Fortune (2013), misrepresentations about the chance of winning can derive from the representativeness heuristic, i.e., a tendency for people to base their judgment of the probability of a particular event on how much it represents the essential features of the parent population or of its generating process (Kahneman et al., 1982), and associated biases. For instance, one of the most documented biases related to gambling is *the* *gambler’s fallacy*, which occurs when individuals believe that even short strings of random events must correspond with their perception of what constitutes randomness, leading to beliefs that particular outcomes are “due” (Tversky and Kahneman, 1971).

On the other hand, a *contaminated* *mindware* as superstitious thinking, i.e., the propensity of having beliefs based on perceiving biased casual relationships between unrelated events (Ninness and Ninness, 1998), can be related to distortions about gambling. Superstition, which appears during childhood and adolescence (Chiesi et al., 2010), is a thinking disposition that can affect reasoning regardless of cognitive abilities (Sá et al., 2005; West et al., 2008). Research and practice with adult pathological gamblers have shown that they have behavioral superstitions in which they associate certain habits with positive gambling results, cognitive superstitions in which they associate specific thought processes with winning, or talismanic superstitions in which they associate good luck charms with winning (Toneatto, 1999).

Taken together, *mindware gap* and *contaminated mindware*, as defined inside the above described dual-process framework, can provide an explanation for the mechanisms under which *gambling-related cognitive distortions* arise. These distortions, e.g., mistaken perceptions of the role of personal ability in gambling, misrepresentations of the chances of winning, false beliefs about the possibility to control or predict gambling outcomes, are deemed important risk factors for pathological gambling in both adults and adolescents. Indeed, high levels of cognitive distortions have been found to be associated with high levels of gambling frequency and to play an important role in the development of problem gambling in adults (Raylu and Oei, 2004; Arcan and Karanci, 2015; see Fortune and Goodie, 2012, for a review). Consistently, cognitive distortions related to gambling predict the frequency of gambling (Donati et al., 2015) and are strong predictors of problem gambling among adolescents (e.g., Taylor et al., 2014; Cosenza and Nigro, 2015; Donati et al., 2015).

Following these premises, we aimed to test a model in which susceptibility to the *gambler’s fallacy* (*mindware gap*) and superstitious thinking (*contaminated mindware*) were associated with gambling frequency and problem gambling through gambling-related cognitive distortions. Indeed, given the importance of cognitive distortions in relation to gambling behavior, it becomes relevant to investigate their possible antecedents in young people. To the best of our knowledge, there are few studies on this topic and, in particular, there is a lack of studies attesting empirically the relationship between probabilistic reasoning and superstition to gambling-related cognitive distortions among adolescents.

We hypothesized that higher susceptibility to the gambler’s fallacy and higher superstitious thinking would be related to higher gambling-related erroneous cognitions. Moreover, since both susceptibility to the gambler’s fallacy and superstitious thinking have been found to be related to gambling behavior among adolescents (e.g., Skoukaskas and Satkeviciute, 2007; Delfabbro et al., 2009; Chiu and Storm, 2010; Donati et al., 2013), we predicted that cognitive distortions related to gambling would mediate the relationship between susceptibility to the gambler’s fallacy and superstitious thinking with gambling frequency and problem gambling. Furthermore, as the frequency of gambling has been found to be linked to the number of problem gambling symptoms (Chiu and Storm, 2010; Derevensky et al., 2010), we predicted that gambling-related cognitive distortions would affect problem gambling also indirectly through gambling frequency. Finally, as probabilistic reasoning biases have been found to be related to superstition (e.g., Kokis et al., 2002; Chiesi et al., 2010), we hypothesized a positive correlation between susceptibility to the gambler’s fallacy and superstitious thinking.

### Methods

#### Participants

Participants included 306 male adolescents (*M*_age_ = 17.2 years, *SD* = 1.5, range: 14–24) who attended high school in Italy (Tuscany). In line with some studies (e.g., Vitaro et al., 2004; Ricijas et al., 2016), we recruited only boys. Indeed, despite the expansion of the gambling industry has modified the male-dominated gambling culture (Dowling, 2013), gender differences in gambling behavior have been reported, indicating that boys are more likely than girls to gamble and to report gambling problems (see Splevins et al., 2010; Calado et al., 2016, for reviews). Written informed assent was provided by students and by the parents if the student was a minor.

#### Measures and Procedure

To measure susceptibility to the gambler’s fallacy, the *Gambler’s Fallacy Task* (GFT, Primi and Chiesi, 2011) was used. It consists of a marble bag game in which participants were asked which outcome was more likely at the next draw after a sequence of five equal outcomes (five blue or five green marbles). In more detail, the task was composed of three different trials in which the proportion of Blue and Green marbles in the bag varied (first trial: 15B and 15G; second trial: 10B and 20G; third trial: 25B and 5G). In total, each participant answered six questions. Summing fallacious answers, we computed a gambler’s fallacy score ranging from 0 to 6, with higher scores corresponding to higher susceptibility to the gambler’s fallacy.

To measure superstitious thinking, the *Superstitious Thinking Scale* (STS, Kokis et al., 2002; Italian version: Chiesi et al., 2010) was used. It is composed of eight Likert-type items using a 5-point scale ranging from *totally false* to *totally true*, yielding a maximum score of 40. Higher scores represent high levels of superstitious thinking. An example of an item is “*The number 13 is unlucky*”. Coefficient alpha for the current sample was satisfactory (α = 0.77).

The *Gambling Related Cognitions Scale* (GRCS; Raylu and Oei, 2004; Italian version: Iliceto et al., 2015) is a self-report scale to assess gambling-related cognitions. It contains twenty-three Likert-type items (using a 7-point scale ranging from *strongly disagree* to *strongly agree*) related to five biases regarding gambling measured by the following subscales: *Gambling Expectancies* (4 items; e.g., “*Having a gamble helps reduce tension and stress*”), *Illusion of Control* (4 items; e.g., “*Specific numbers and colors can help increase my chances of winning*”), *Predictive Control* (6 items; e.g., “*When I have a win once, I will definitely win again*”), *Inability to Stop Gambling* (5 items; e.g., “*It is difficult to stop gambling as I am so out of control*”), and *Interpretative Bias* (4 items; e.g., “*Relating my losses to bad luck and bad circumstances makes me continue gambling*”). The scale was previously found to have adequate validity and reliability among adolescents (e.g., Taylor et al., 2014; Donati et al., 2015). The coefficient alpha for the current sample was satisfactory (α = 0.89). The GRCS subscale scores as well as the GRCS total score, obtained by summing the score for each item, were calculated. However, following the suggestion that only the total score for the GRCS should be used with adolescents (Taylor et al., 2014), the total score was used in the path model.

Gambling behavior was measured through the *South Oaks Gambling Screen-Revised for Adolescents* (SOGS-RA; Winters et al., 1993; Italian version: Colasante et al., 2014). This is one of the most widely instrument to measure problem gambling with adolescents (see Edgren et al., 2016, for a review), and its effectiveness has been attested by applying Item Response Theory (Chiesi et al., 2013). The scale is composed of two sections using the last year gambling behavior. In the first one, participants were asked to indicate the frequency of gambling (Never = 0, Less Than Monthly = 1, Monthly = 2, Weekly = 3, and Daily = 4) among a list of eleven gambling activities including: Playing cards for money, coin tosses for money, bets on games of personal skill, bets on sports teams, bets on horse or dog races, bingo, dice games for money, slot machines, scratch-cards, lotteries, and on-line games. Considering responses to this section, participants can be classified into *non-gamblers* (no gambling behavior) and *gamblers* (gambling on at least one activity) (Welte et al., 2009). Moreover, among gamblers, *non-regular gamblers* (i.e., those who participated from less than monthly to less than weekly in at least one gambling activity) and *regular gamblers* (i.e., those who participated weekly or daily in at least one gambling activity) can be identified (Winters et al., 1993). Finally, a total score of gambling frequency (range: 0–44) can be obtained by summing the responses for each gambling activity (Wickwire et al., 2007). The second section consists of 12 items related to the *Diagnostic and Statistical Manual of Mental Disorders* (III edition revised) criteria for pathological gambling (American Psychiatric Association, 1987). An example is: “*In the past 12 months, how often have you gone back another day to try to win back money that you lost?*”. All items require dichotomous answers (i.e., yes or no) except the first item, which has a 4-point response scale (never, some of the time, most of the time, every time), and it is dichotomized (i.e., never/some of the time or most of the time/every time) in the scoring phase. A single composite score was computed summing the responses for each item of the second section. The total SOGS-RA score, indicative of the number of problem gambling symptoms, was used as dependent variable (range: 0–12), in line with previous studies (e.g., Wickwire et al., 2007, 2010). Finally, according to the narrow criterion (Winters et al., 1995), different categories of gamblers were identified: *Non-problem gamblers* (i.e., SOGS-RA scores from 0 to 1), *at-risk gamblers* (i.e., SOGS-RA scores from 2 to 3), and *problem gamblers* (i.e., SOGS-RA scores of 4 or more).

The above-described scales were administered in the classrooms and students were required to work individually. Teachers were not present during the administration of the scales, which required approximately 40 min.

### Results

Results showed that 16% of the participants had never gambled. Then, we performed the analyses on adolescent gamblers, i.e., the 254 respondents who affirmed having gambled at least once during the last year. Among them, 66% were *non-regular gamblers* (*n* = 86), and 34% were *regular gamblers* (*n* = 168). The most common activities were scratch-cards (74%), sport bets (62%), and cards for money (47%), while the least engaged in activities were dice games for money (7%), bets on coin tosses (8%), and bets on horse or dog races (9%). Considering the score of the second section of the SOGS-RA, 75% (*n* = 190) of the respondents were *non-problem gamblers*, 19% (*n* = 48) *at-risk gamblers*, and 6% (*n* = 16) *problem gamblers*. Descriptive statistics of GFT, STS, GRCS, and the SOGS-RA based upon gambling frequency are displayed in **Table 1**, while descriptive statistics of the scales for the entire sample are reported in **Table 2**.

**Table 1:** Descriptive statistics for gambler’s fallacy, superstitious thinking, gambling-related cognitive distortions – the GRCS total score and the subscale scores – and problem gambling for non-regular gamblers (*n* = 86) and Regular gamblers (*n* = 168).

	Type of gamblers based upon gambling frequency				
Dependent variable	Non-regular	Regular	*t* (df)	*p*	Cohen’s *d*
	gamblers *M* (*SD*)	gamblers *M* (*SD*)			
Gambler’s fallacy	4.56 (*1.73*)	5.19 (*1.44*)	-2.74 (*252*)	<0.01	0.40
Superstitious thinking	18.49 (*6.25*)	21.36 (*7.10*)	-3.30 (*252*)	<0.01	0.43
Gambling related cognitive distortions – Total score	35.68 (*12.31*)	48.66 (*20.36*)	-6.32 (*252*)	<0.001	0.77
*Gambling Expectancies*	5.39 (*2.08*)	8.55 (*4.64*)	-7.49 (*252*)	<0.001	0.88
*Illusion of Control*	5.35 (*2.50*)	7.07 (*3.99*)	-4.20 (*252*)	<0.001	0.70
*Predictive Control*	11.65 (*5.67*)	14.42 (*6.36*)	-3.53 (*252*)	<0.001	0.46
*Inability to Stop Gambling*	6.54 (*2.68*)	8.46 (*2.57*)	-3.87 (*252*)	<0.001	0.73
*Interpretative Bias*	6.75 (*3.75*)	10.16 (*5.44*)	-5.86 (*252*)	<0.001	0.73
Problem gambling	0.60 (*1.06*)	1.81 (*2.07*)	-6.20 (*252*)	<0.001	0.74

**Table 2:** Means, standard deviations, and correlations among gambler’s fallacy, superstitious thinking, gambling-related cognitive distortions – the GRCS total score and the subscale scores -, gambling frequency, and problem gambling.

	1	2	3	4	5	6	7	8	9	10
1 Gambler’s fallacy	–									
2 Superstitious thinking	0.21**	–								
3 Gambling-related cognitive distortions–total score	0.24***	0.28***	–							
4 *Gambling Expectancies*	0.20**	0.26***	0.82***	–						
5 *Illusion of Control*	0.25***	0.39***	0.72***	0.56***	–					
6 *Predictive Control*	0.20**	0.22***	0.83***	0.54***	0.55***	–				
7 *Inability to Stop Gambling*	0.19*	0.16*	0.72***	0.57***	0.38***	0.43***	–			
8 *Interpretative Bias*	0.10	0.12	0.79***	0.61***	0.42***	0.53***	0.49***	–		
9 Gambling frequency	0.15*	0.19**	0.50***	0.56***	0.30***	0.26***	0.49***	0.41***	–	
10 Problem gambling	0.11	0.03	0.54***	0.47***	0.25***	0.35***	0.53***	0.50***	0.54***	–
*M* (*SD*)	4.93 (*1.57*)	19.46 (*6.68*)	40.07 (*16.65*)	6.46 (*3.51*)	5.93 (*3.19*)	12.59 (*6.04*)	7.19 (*3.86*)	7.91 (*4.67*)	5.45 (*4.56*)	1.01 (*1.58*)

^∗^*p < 0.05, ^∗∗^*p* < 0.01, ^∗∗∗^*p* < 0.001*.

As reported in **Table 1**, results showed that *regular gamblers* were more susceptible to the gambler’s fallacy, had higher levels of superstitious thinking and gambling related cognitive distortions, and reported more problem gambling symptoms than *non-regular gamblers*.

Then, we computed Pearson correlations to investigate the relationships among susceptibility to the gambler’s fallacy, superstitious thinking, gambling-related cognitive distortions – the GRCS total score and the subscale scores-, gambling frequency, and problem gambling.

As shown in **Table 2**, gambling-related cognitive distortions were significantly and positively correlated both with susceptibility to the gambler’s fallacy and superstitious thinking. In detail, with the exception of *Interpretative Bias*, all the five cognitive distortions were related to susceptibility to the gambler’s fallacy and superstitious thinking, especially *Illusion of Control*, which shows, respectively, moderate and high correlations with the two variables. In addition, gambling-related cognitive distortions were significantly and positively correlated both with gambling frequency and problem gambling. Looking at GRCS subscales correlations, results indicated moderate and high correlations, with the highest between gambling frequency and *Gambling Expectancies*, while, *Inability to Stop Gambling* showed the highest Pearson coefficient value in the association with problem gambling. The results also showed that susceptibility to the gambler’s fallacy was significantly and positively correlated with superstitious thinking, and both these variables were significantly and positively correlated with gambling frequency. The correlations between problem gambling were not significant. Finally, gambling frequency resulted to be significantly and positively correlated with problem gambling.

**FIGURE 1:**
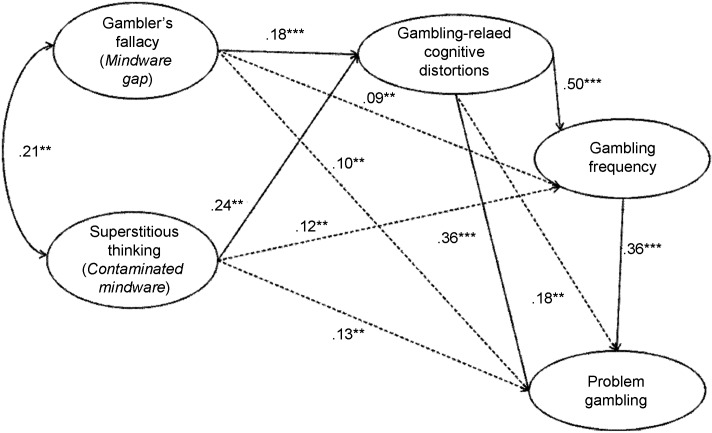
Model of problem gambling with standardized parameters (significant path coefficient ^∗∗∗^ at the 0.001 level, ^∗∗^ at the 0.01 level). Dotted lines represent indirect effects, while continuous lines indicate direct effects.

To investigate our hypothesis on the mechanisms underlying the relationships among these variables, we conducted a path analysis with AMOS using maximum likelihood estimation. The model included susceptibility to the gambler’s fallacy and superstitious thinking as gambling-related cognitions’ antecedents, and gambling-related cognitive distortions as antecedents of gambling frequency and problem gambling, which was directly affected by gambling frequency (**Figure 1**). Several goodness-of-fit indices were used to test the adequacy of the model: The Comparative Fit Index (CFI; Bentler, 1990), the Tuker-Lewis index (TLI; Tucker and Lewis, 1973), and the Root Mean Square Error of Approximation (RMSEA; Steiger and Lind, 1980). CFI and TLI values equal to.90 or greater (Tucker and Lewis, 1973; Bentler, 1990) and RMSEA values of.08 or below (Steiger and Lind, 1980) were considered as indices of adequate fit.

The hypothesized model showed a good fit to the data (CFI = 0.98, TLI = 0.94, RMSEA = 0.07). All coefficients were statistically significant and in the expected directions. Specifically, results revealed that susceptibility to the gambler’s fallacy and superstitious thinking had significant direct positive effects on gambling-related cognitive distortions. Gambling-related cognitive distortions were directly and positively related to gambling frequency as well as problem gambling, and gambling frequency had a direct positive effect on problem gambling. Moreover, both susceptibility to the gambler’s fallacy and superstitious thinking had significant indirect effects on gambling frequency, respectively 0.09 (*p* < 0.01) and 0.12 (*p* < 0.01), and on problem gambling, respectively 0.10 (*p* < 0.01) and 0.13 (*p* < 0.01). A significant indirect effect of 0.18 (*p* < 0.01) was also found between gambling-related cognitions and problem gambling Finally, a significant positive covariation was found between susceptibility to the gambler’s fallacy and superstitious thinking.

### Discussion

The aim of this study was to test the adequacy of a model explaining the relationship between susceptibility to the gambler’s fallacy, superstitious thinking, gambling-related cognitive distortions, and gambling behavior among adolescents. In line with the predictions, findings revealed that the tendency to commit the gambler’s fallacy and to be superstitious affects distorted cognitions about gambling. More specifically, higher susceptibility to commit the gambler’s fallacy and higher superstitious thinking were related to greater levels of gambling-related cognitive distortions. Moreover, our model showed that cognitive distortions on gambling mediate the relationship between susceptibility to the gambler’s fallacy and superstitious thinking with gambling behavior.

From a theoretical point of view, our findings confirm that there may be a cognitive-psychological mechanism through which faulty beliefs about gambling develop. In particular, results are consistent with Toplak et al.’s (2007) suggestions about the existence of two cognitive processes that affect problematic gambling behavior in adults–following a dual-process perspective on cognitive functioning (Stanovich, 2004)– i.e., difficulties in dealing with probability (*mindware gap*) and belief in superstition and luck (*contaminated* *mindware*).

Compared to previous research with adolescents, our study expands the current knowledge by suggesting that gambler’s fallacy and superstitious thinking are related to gambling-related distortions in adolescence. Furthermore, this study suggests that the relationship between gambler’s fallacy and problem gambling (e.g., Skoukaskas and Satkeviciute, 2007; Delfabbro et al., 2009; Donati et al., 2013), and the association of superstitious thinking with problem gambling (e.g., Chiu and Storm, 2010; Donati et al., 2013) can be explained by taking into account the mediating role of gambling-related cognitive distortions. In other words, adolescents more prone to mistaken perceptions of probability and with the tendency to adhere to superstitious beliefs are susceptible to cognitive distortions related to gambling. As such, they are particularly at risk since they have a greater likelihood of gambling with high frequency and developing gambling problems.

Although this model has been verified with a relatively small number of adolescent male gamblers, practical implications can be derived from the study. Indeed, our model can represent a theoretical based framework from which developing theory-driven interventions oriented to youth. Specifically, focusing on these findings, a program aimed to modify gambling-related cognitive distortions can be developed. This was the aim of Study 2.

## Study 2

Among the guidelines published by the Society for Prevention Research for the development of effective preventive interventions (Flay et al., 2005), there is the desirable standard that a clear theory of causal mechanism of the change promoted by the intervention should be stated. In particular, it would be important that the preventive program would be informed by theory and prior empirical analyses on antecedents and predictors of outcomes. Indeed, the systematic reviews conducted on the preventive interventions developed with adolescents in the school setting (Ladouceur et al., 2013; St-Pierre et al., 2015; Keen et al., 2016) agree in recognizing that many of the existing prevention programs have been developed in absence of a clear theoretical framework describing the expected causal mechanisms by which the programs would exert their effect.

Following this premise, once tested the adequacy of our model on gambling in Study 1, the goal of Study 2 was to develop and verify the effectiveness of a universal preventive intervention addressed to general samples of youth, regardless of risk or gambling status (Ladouceur et al., 2013; Keen et al., 2016) aimed to reduce gambling-related cognitive distortions by acting on probabilistic reasoning errors (*mindware gap*) and superstitious thinking (*contaminated* *mindware*). Additionally, moving from the theoretical guideline for which the main purpose of any prevention program should be to reduce the incidence of the potential problem (Ladouceur et al., 2013), we aimed to obtain behavioral changes related to gambling frequency, which is an antecedent of problem gambling (Chiu and Storm, 2010; Derevensky et al., 2010). Finally, due the “preventive” nature of the current intervention, reducing the incidence of problem gambling was outside our goals. In sum, following St-Pierre et al.’s (2015) classification framework, we developed a “*gambling-specific psychoeducational and skills training prevention program*” to reduce the erroneous cognitions on gambling acting on gambling-related knowledge, beliefs, attitudes, and skills as well as the awareness about the nature of gambling, knowing that all these factors may impact on adolescents’ gambling habits.

In evaluating the effectiveness of the proposed intervention, we also wanted to take into account some relevant methodological issues. First of all, although a short-term change of gambling-erroneous cognitions have been obtained in several of these preventive initiatives (e.g., Ferland et al., 2002, 2005; Capitanucci et al., 2010; Williams et al., 2010; Donati et al., 2014; Huic et al., 2017), only few of them verified the stability of these effects over time (Gaboury and Ladouceur, 1993; Capitanucci et al., 2010; Donati et al., 2014). Thus, to provide evidence of the strength and stability of the change in the current intervention program, we assessed the short-term and long-term effects on gambling-related cognitions and also the long-term effects on gambling behavior. Secondly, we employed scales (i.e., SOGS-RA, GRCS, GFT, and STS) that were previously analyzed for their psychometric properties (see Study 1 for a detailed description). Indeed, the majority of the gambling intervention programs have not used psychometrically good measurement instruments to assess the variables of interest despite their obvious necessity (Ladouceur et al., 2013). Finally, we employed an experimental design in which we verified the baseline equivalence of the experimental group and control group for the targeted variables of our intervention. Even in this case, with few exceptions (Williams et al., 2010; Donati et al., 2014), the baseline equivalence between the experimental and control groups has not been tested in past studies.

### Methods

#### Participants

Participants were 34 male high school students (*M*_age_ = 16.80, *SD* = 1.04, range: 15–19) enrolled in a public high school in Tuscany (Italy). From the available schools in the area, one school was randomly selected. Subsequently, the school’s principal was contacted, apprised of the issue of adolescent problem gambling to generate support for the research, and he was presented with the project. Once the school agreed to participate, the detailed study protocol was approved by the institutional review board of the school. Written informed consent was requested from students (or their parents, if they were minors), assuring them that the data would be handled confidentially. The research was conducted during school time and all students invited to participate agreed to do so. We chose a specific sample as it seems pertinent to deliver interventions to small groups of students that are homogenous in terms of risk factors, gambling habits, gender, and age (Ladouceur et al., 2013).

#### Measures

In line with Study 1, participants were administered the GFT, the STS, the GRCS, and the SOGS-RA (see Study 1 for description and scoring).

#### Procedure and Design

To evaluate changes in the dimensions considered in the study over time as a function of treatment condition, an experimental design was conducted with two groups (Training vs. No Training) and three measurements (pre-test, post-test, and follow-up sessions). Classes were randomly assigned to the Training and No Training conditions. The Training group consisted of 16 students (*M*_age_ = 16.99, *SD* = 1.20) and the No Training group consisted of 18 students (*M*_age_ = 16.63, *SD* = 0.89). For the Training group, participation involved filling out the above described scales before the intervention (pre-test), receiving training activities, filling out the GRCS after intervention (post-test), and then compiling the GRCS and the SOGS-RA six months (school break over the summer occurred during this interval) after the intervention has ended (follow-up). The pre-test, post-test, and follow-up questionnaires were administered also to the No Training group. Nevertheless, while the Training group received the intervention, the No Training group continued with usual school activity.

In the pre-test, post-test, and follow-up sessions, the scales were administered within the classrooms, and students were required to work individually. Teachers were not present during the administration of the scales. Administration of the instruments required approximately 40 min for the pre-test session, 15 min for the post-test session, and 25 min for the follow-up session. The Training group attended the intervention approximately 2 weeks after the pre-test, and the post-test was administrated 1 week after the end of the intervention and 5 weeks after pre-test data were collected. Few days after the follow-up session, a final meeting took place during which all the participants were given a feedback about the research and thanked for their participation.

#### The Intervention

Our intervention activities were based upon the model tested in Study 1. In detail, as cognitive distortions on gambling are affected by problems regarding *mindware gap*, i.e., probabilistic reasoning errors, and *contaminated mindware*, i.e., superstitious thinking, we wanted to implement activities in which adolescents could reinforce their ability to recognize biases in reasoning with randomness and could reflect about the irrationality of superstitions. Specifically, as for the *mindware gap*, activities focused on: Randomness with a series of coin tosses, independent random events employing a 40 cards desk, independence with equally likely and non-equally likely events represented with different colored paper sheets, gambler’s fallacy in no-gambling and gambling contexts, and probabilistic reasoning in fictitious gambling situations. Regarding the *contaminated mindware*, participants were told about the superstition meaning and the lack of cause-effect relationship between a supposed event bringing bad or good luck and the supposed positive or negative event occurred referring to common superstitions. Then, referring more specifically to the relationship between superstition and gambling, several examples were presented about susceptibility to superstitious conditioning in gambling activities and the absence of a causal relationship between superstitious thoughts (e.g., the belief in lucky numbers) and gambling outcomes.

Concerning the training techniques, we integrated a mixed set of techniques including activities with random events generators, Power-Point presentations, and collective discussions. As for the methodology, each didactic unit included exercises in which students had to apply the learned ability/concept, and then they had to use the learned ability referring to fictitious gambling situations. In that way, training activities were aimed to promote the generalization of the proposed contents in real-life contexts. Concerning the procedure, each activity was implemented using a specific sequence: Initial instructions by the trainer, running the activity by the students, interactive discussion and synthesis of the contents, delivery of summary sheets to the students.

The intervention included two didactic units implemented in class, during the normal school time conducted by a developmental psychologist expert in the field of adolescent gambling research with a couple of operators belonging to the addiction unit of the socio-territorial service. Teachers were not present during the administration of the training program. Each didactic unit lasted about 2 h and were presented in a 2 week period (one per week).

### Results

Results showed that 85% of the participants (*n* = 29) affirmed having gambled at least once during the last year. Among them, 76% were *non-regular gamblers*, and 24% were *regular gamblers*. The most common activities were scratch-tickets (62%), sport bets (41%), and cards for money (23%). Considering the score of the second section of the SOGS-RA, 81% (*n* = 23) of the respondents were *non-problem gamblers*, 12% (*n* = 4) *at-risk gamblers*, and 6% (*n* = 2) *problem gamblers*.

Preliminarily, we tested the baseline equivalence of the Training and No Training groups for age and the targeted variables of our intervention. No significant differences were found between the two groups concerning age (*p* = 0.316), susceptibility to the gambler’s fallacy (*p* = 0.111), and superstitious thinking (*p* = 0.661). Then, we analyzed the short-term efficacy of the intervention conducting a Mixed 2 × 2 ANOVA with Time (pre- and post-test) as within factor, Group (Training and No Training) as between factor, and gambling-related cognitive distortions as dependent variable.

**FIGURE 2:**
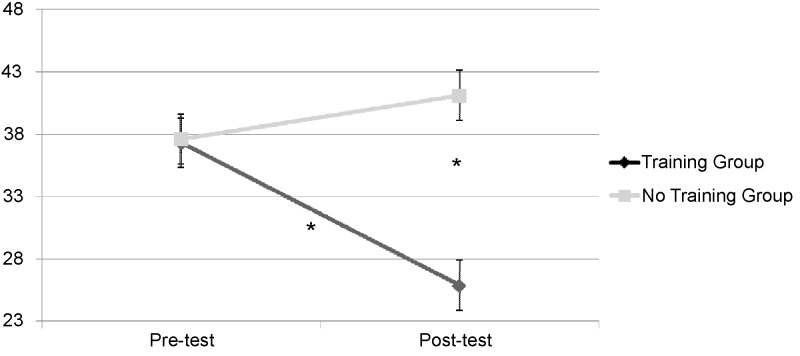
Gambling-related cognitive distortions by Time (pre-test and post-test) and group (Training and No Training). *^∗^p < 0.05*.

A significant Time × Group interaction was found [*F*(1,32) = 4.25, *p* < 0.05, $\eta_{p}^{2}$ = 0.117]. *Post hoc* *t-*tests showed the interaction effects to be due to significant changes from pre-test to post-test in the Training group but not in the No Training group. Specifically, in the Training group there was a significant reduction of gambling-related cognitive distortions [*t*(15) = 2.78, *p* < 0.05, Cohen’s *d* = 0.69) from pre-test (*M* = 37.31, *SD* = 17.53) to post-test (*M* = 25.88, *SD* = 5.25), while no significant changes occurred in the No Training group [*t*(17) = -0.61, *p* = 0.552] from pre-test (*M* = 37.61, *SD* = 10.95) to post-test (*M* = 41.11, *SD* = 26.43). Moreover, the two groups resulted to be significantly different for gambling-related cognitive distortions at the post-test [*t*(32) = -2.26, *p* < 0.05, Cohen’s *d* = 0.80]. The Training group resulted to have lower levels of erroneous cognitions about gambling compared with the No Training group, while at the pre-test they have an equivalent level (**Figure 2**).

To verify the stability of the short-term effects over time, for the Training participants, we compared post-test and follow-up scores of gambling-related cognitive distortions. In detail, using paired *t*-tests, we compared post-test scores with the follow-up ones. Results showed no significant differences [*t*(15) = -0.29, *p* = 0.780] suggesting the permanence of the intervention effects over time for gambling-related cognitive distortions from post-test (*M* = 25.88, *SD* = 5.25) to follow-up (*M* = 26.31, *SD* = 6.66).

Subsequently, to verify whether the intervention had a decrementing effect on adolescent self-reported gambling behavior, a Mixed 2 × 2 ANOVA with Time (pre- and follow-up) as within factor, Group (Training and No Training) as between factor, and gambling frequency as dependent variable, was conducted. A non-significant Time × Group interaction was found [*F*(1,32) = 1.70, *p* = 0.201]. Nonetheless, since the sample size was small and important effect might be non-significant (i.e., Type II errors might be made), we looked at the effect size ($\eta_{p}^{2}$ = 0.05), which suggested that a small effect was obtained. As such, *post hoc* *t*-tests showed significant changes from pre- to follow-up in the Training group but not in the No Training group. Specifically, in the Training group there was a significant and medium size change of gambling frequency [*t*(15) = 2.95, *p* < 0.05, Cohen’s *d* = 0.73], suggesting a reduction of gambling frequency from pre-test (*M* = 3.69, *SD* = 4.96) to follow-up (*M* = 1.50, *SD* = 2.53). On the contrary, no significant changes occurred in the No Training group [*t*(17) = 0.04, *p* = 0.969] from pre-test (*M* = 3.94, *SD* = 4.45) to follow-up (*M* = 3.89, *SD* = 5.22).

### Discussion

Following the results of Study 1, the aim of the present study was to develop and evaluate a preventive intervention which would be able to modify erroneous cognitions about gambling by acting on probabilistic reasoning biases (*mindware gap*) and superstitious thinking (*contaminated* *mindware*). Findings showed that the intervention produced the hypothesized effects in the short-term as participants who attended the training program reduced their gambling-related cognitive distortions, while the participants who did not follow the training program did not show a significant change from pre-test to post-test. This finding is of particular importance since research has generally provided evidence of a resistance to change for probabilistic reasoning biases (for a summary of the literature, see e.g., Gilovich et al., 2002; specifically, for adolescents, see Klaczynski, 2004). Additionally, the above described short-term results for gambling erroneous distortions were found to be stable after 6 months by the end of the intervention (i.e., the post-test mean scores did not differ significantly from the follow-up), indicating a substantial persistence of the effects over a period of six months for participants attending the training program.

Concerning the effects on gambling behavior, whereas some previous studies reported no behavioral changes despite improvements in knowledge and the reduction of cognitive errors (Gaboury and Ladouceur, 1993; Ferland et al., 2005; Turner et al., 2008; Huic et al., 2017), some changes were produced in gambling behavior. Specifically, in line with previous studies (Williams, 2002; Donati et al., 2014) we observed that only adolescents who attended the training program reduce their gambling frequency from pre-test to follow-up.

Finally, the methodological strengths of the current study, i.e., having tested short-and long-term effect of the intervention, having used effective instruments to measure the variables of interest, and having tested the baseline equivalence of the experimental and control group, attest to the worth and utility of the proposed intervention.

In sum, the current study provided evidence about the effectiveness of an intervention based upon an evidence-based theoretical model referring to the dual-process theoretical framework.

## General Discussion

The systematic reviews (Ladouceur et al., 2013; St-Pierre et al., 2015; Keen et al., 2016) conducted on the preventive interventions developed with adolescents in the school setting agree in recognizing that many of the existing prevention programs have been developed in absence of a clear theoretical framework describing the expected causal mechanisms by which the programs would exert their effect. Overcoming the limitations of the previous studies, this work proposed and tested the effectiveness of a gambling preventive intervention with adolescents after having previously verified the adequacy of a theoretical model explaining adolescent gambling involvement. With respect to the application of dual-process theories on cognitive functioning in the prediction of gambling behavior (Toplak et al., 2007), our model proposed that susceptibility to the gambler’s fallacy and superstitious thinking (respectively, *mindware gap* and *contaminated mindware*, according to the dual-process theory) were the predictor variables of gambling-related distorted cognitions in adolescence, while gambling frequency and problem gambling were the outcome variables. This study supports the results suggested by Clark (2010) that the high propensity for individuals commit mistakes in reasoning and judgment makes them particularly vulnerable to adhere and maintain cognitive distortions related to gambling. With specific reference to the practical implications of the model, our results provided a theoretically grounded model useful not only to explain gambling-related cognitions but also to develop interventions to modify them.

More broadly speaking, this work showed that dual-process theory of cognition (see Evans and Stanovich, 2013; for a review) can be used as conceptual framework to explain and prevent gambling behavior in adolescence. There have been only few attempts to apply it to adolescents, with few exceptions (see Klaczynski, 2004, for the employment of this theory to explain adolescent social and cognitive development), and little research has been conducted in order to investigate its application in the field of adolescent health behavior. Nevertheless, it has been suggested the utility of dual-process theories in explaining and predicting many types of health behaviors in adolescence as they involve both analytic and heuristics processing (Gibbons et al., 2009). Thus, the present studies provided some empirical support about the applicability of dual-process theories to explain and modify gambling-related erroneous cognition and gambling behavior, in some extent. More in detail, the intervention we developed following the dual-process model resulted was effective in reducing gambling-related erroneous cognitions in the short-term and in producing a stable change of these cognitions in the long-term. Additionally, there was a transfer of learning about gambling-related cognitions onto gambling behavior resulting in a reduction of gambling frequency after six months by the end of the intervention. Nonetheless, the medium effect size of this difference confirms the existing difficulties in changing gambling behavior among adolescents through educational interventions (Keen et al., 2016).

Concerning this point, the effectiveness of this intervention in reducing an important risk factor for maladaptive gambling behavior (i.e., gambling-erroneous cognitions) is very important as it has been showed that preventive programs that obtain change in risk and protective factors are more successful than programs showing behavior change (Foxcroft and Tsertsvadze, 2012). Moreover, these kind of programs focused on changing specific correlates of maladaptive gambling behavior may have effects that extend to other health behaviors (Hawkins et al., 2015).

Whereas this work has a number of strengths, including the evaluation of a theoretical model then linked to a gambling preventive intervention, and the use of good psychometric instruments, there are some limitations to take into account. First, as our work was conducted with boys attending Italian public high school, caution has to be paid about the generalizability of the present results. Future studies should be conducted in order to test the adequacy of the theoretical model with broader samples of adolescents, for instance including also girls. Moreover, although the descriptive data about gambling behavior were in line with previous data with adolescent males (e.g., Gupta et al., 2004; Olason et al., 2006), the small number of participants in Study 2 limits the impact of the current results concerning the developed preventive intervention. Future studies should be conducted with wider samples in order to evaluate the effectiveness of the intervention based on gambling behavior and severity. Finally, as research indicates that various and different factors increase the likelihood of problem gambling in adolescents (e.g., Donati et al., 2013; Cosenza and Nigro, 2015), it should be important to investigate theoretical models taking into account other variables in addition to susceptibility to the gambler’s fallacy and superstitious thinking as predictors of gambling-erroneous cognitions.

## Ethics Statement

This study was carried out in accordance with the APA recommendations of psychology deontology with written informed consent from all subjects. All subjects gave written informed consent in accordance with the Declaration of Helsinki. The protocol was approved by the ethical committees of each school.

## Author Contributions

MD developed the theoretical model and constructed the intervention, which she conducted in the school setting. FC collaborated in the data analysis and revised the preliminary versions of the paper. AI promoted the research project among the schools and contacted the school headmasters. AM and FF participated in data interpretation. CP supervised the entire research project and gave her contribution in the theoretical and practical discussion of the results.

## Conflict of Interest Statement

The authors declare that the research was conducted in the absence of any commercial or financial relationships that could be construed as a potential conflict of interest.
